# Light-driven ammonia electrooxidation via carbon nitride–ruthenium molecular interfaces

**DOI:** 10.3762/bjnano.17.61

**Published:** 2026-07-08

**Authors:** Jan Holub, Pablo Jiménez-Calvo

**Affiliations:** 1 Group of Coordination Chemistry, University of Chemistry and Technology, Prague (UCT, Prague); Department of Inorganic Chemistry, Technická 5, 166 28, Prague, Czech Republic; 2 Chemistry of Thin Film Materials, Materials Chemistry Section, Friedrich-Alexander-Universität Erlangen-Nürnberg, Cauerstraße 3, IZNF, Erlangen 91058, Germany; 3 Friedrich-Alexander-Universität Erlangen-Nürnberg, Profile Center Solar (FAU Solar), Freyeslebenstraße 1, 91058 Erlangen, Germany; 4 Department of Colloid Chemistry, Max-Planck-Institute of Colloids and Interfaces, Am Mühlenberg 1, 14476 Potsdam, Germany

**Keywords:** ammonia oxidation, carbon nitride, hybrid semiconductor–molecular interfaces, molecular ruthenium catalysts, photoelectrocatalysis

## Abstract

The homogeneous–heterogeneous catalysis gap remains an unresolved challenge in solar fuels research. Molecular catalysts offer unique selectivity and mechanistic transparency but suffer from poor electrode contact and limited recyclability, while heterogeneous semiconductors provide scalable light harvesting but lack precisely defined active sites. Anchoring molecular ruthenium (Ru) catalysts onto heterogeneous semiconductors, like carbon nitride (C_3_N_4_), offers a chemically rational strategy to bridge this gap, yielding hybrid photoelectrodes capable of driving ammonia oxidation, a reaction of growing importance as a sustainable hydrogen carrier. Inspired by natural photosynthesis, in which a light-harvesting antenna is spatially coupled to a multielectron catalytic centre, the proposed hybrid system assigns distinct and complementary roles to each component: C_3_N_4_ absorbs visible light, separates charge carriers, and provides a structurally tunable aromatic surface, while the metal complexes, e.g., RuBda, RuTda, or RuTpyBpy, accept photogenerated holes and drive the demanding six-electron oxidation of ammonia through well-defined coordination chemistry. Two anchoring strategies, covalent amide bond formation exploiting the surface amine groups of C_3_N_4_, and non-covalent π–π and C–H···π interactions mediated by pyrene-functionalized ligands, are presented as complementary rather than competing routes to the heterointerface, each controlling surface density, electronic coupling, and catalyst stability differently. This perspective article examines how the structural diversity of the C_3_N_4_ allotropes, spanning semicrystalline polymeric C_3_N_4_, highly ordered poly(heptazine imide), and high-surface-area amorphous sulfur-doped C_3_N_4_, offers a tunable platform for optimizing charge carrier dynamics at the hybrid interface. Finally, photoelectrocatalysis is the enabling configuration: Simultaneous illumination and electrochemical bias reduce the thermodynamic penalty, suppress charge recombination, and provide independent control over product selectivity. Despite available materials, precedent reactions, and compelling mechanistic rationale, no study to date has reported photoelectrocatalytic ammonia oxidation at a C_3_N_4_–Ru hybrid photoelectrode, this gap is the motivation and the central argument of this perspective.

## Perspective

### A) The energy challenge and the catalysis gap

The global transition away from fossil fuels demands new paradigms for how chemical energy is stored and released. At the heart of this challenge lies catalysis. For decades, it has been divided into two families, yet their integration is already bearing fruit. In water oxidation catalysis, molecular-semiconductor hybrids have demonstrated performance in electrocatalytic and photocatalytic systems, as proof of concept for bridging homogeneous precision with heterogeneous scalability. Homogeneous catalysts (molecular complexes in solution) operate with exquisite precision, that is, well-defined active sites, traceable mechanisms, and selective tunability achievable atom by atom via ligand design. Heterogeneous catalysts (metal oxides, nanoparticles, and carbon supports) are manufactured into electrodes or serve as the electrode itself, industrially scalable, but with poorly understood active sites and inactive bulk material [1–5].

In practice, however, bridging these two families is not straightforward. A homogeneous catalyst dissolved in solution faces a fundamental limitation: Only a tiny fraction of dissolved molecules is ever close enough to an electrode surface to participate in electron transfer at any given moment. The rest drifts idly in solution, increasing precious metal loading without contributing to the reaction. Heterogeneous catalysts solve the electrode contact problem but introduce their own: In a thick particle film, only the outermost surface layer is electrochemically accessible, while the underlying bulk is not [6–7]. For reactions that require both a light-absorbing semiconductor and a chemically active catalytic centre, as is the case in solar fuel production, neither paradigm alone is sufficient. What is needed is a hybrid architecture that positions a well-defined molecular catalyst directly at the surface of a photoactive solid, combining the selectivity of homogeneous catalysis with the processability and light-harvesting capacity of heterogeneous materials [1–5,8].

This perspective focuses on the well-known combination regarding water oxidation, that is, C_3_N_4_ semiconductors with molecular Ru-based catalysts as one promising multicomposite hybrid strategy. C_3_N_4_ acts as the light absorber and charge-conducting scaffold, functionally analogous to a photosensitizer, while the Ru complex serves as the molecular catalytic centre and light absorber too. Transferring this synergistic photoactive interface concept into photoelectrocatalysis for ammonia oxidation, a sustainable hydrogen carrier, remains an open and compelling opportunity [8].

### B) Learning from nature: photosynthesis as the conceptual template

**Figure 1:**
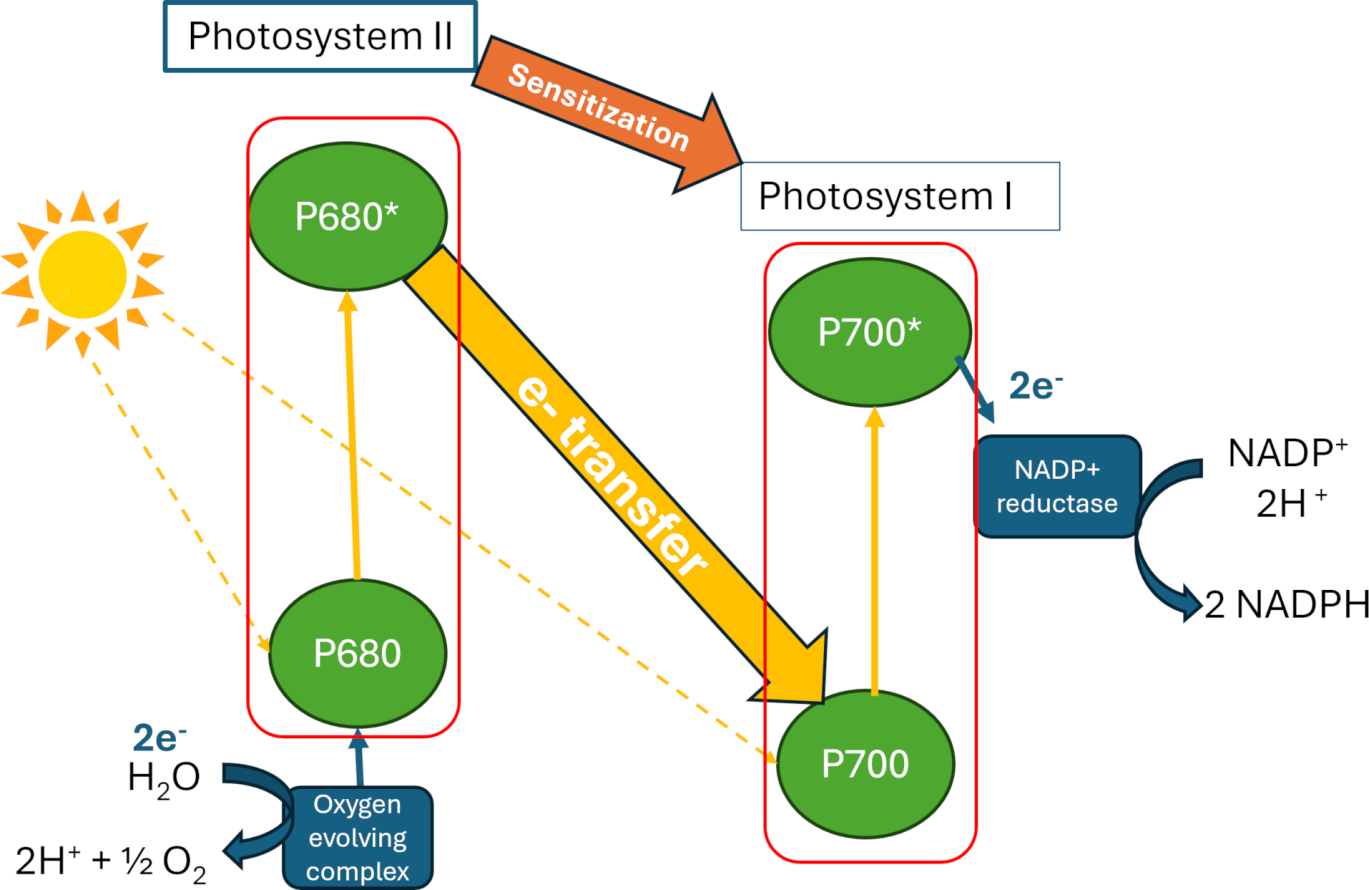
Scheme of photosynthesis, focusing on the electron transfer and the sensitisation effect.

The photoactive system took inspiration from natural photosynthesis (Figure 1). Plants, algae, and cyanobacteria optimise the conversion of sunlight into chemical energy, and the architecture they arrived at contains design principles of elegance that chemists are only beginning to replicate [9]. In the thylakoid membrane of plant chloroplasts, light harvesting begins with antenna complexes, assemblies of pigment molecules, chlorophylls and carotenoids, arranged in a precisely ordered protein scaffold. These antenna pigments absorb photons across a broad range of the visible spectrum and funnel the resulting electronic excitation energy, with near-unity quantum efficiency, to a specialised reaction centre. This energy funnelling process is called photosensitization: The antenna molecules do not themselves drive the chemistry, but they extend the spectral range and photon capture cross section of the system, ensuring that the reaction centre receives a concentrated supply of excitation energy even under low-light conditions [9–10].

At the reaction centre of Photosystem II, the absorbed excitation drives the oxidation of water (a thermodynamically demanding four-electron, four-proton process) at an inorganic cluster containing four manganese ions and one calcium ion (Mn_4_CaO_5_). The electrons extracted from water are passed along the electron transport chain to Photosystem I, where they reduce biological reductant NADP^+^ to NADPH, which powers carbon fixation. The protons liberated by water oxidation are used to drive ATP synthesis, and the oxygen released is the molecular oxygen that constitutes Earth’s atmosphere. The entire system is spatially organised within the membrane so that charge separation is maintained over nanometer distances and millisecond timescales, preventing the wasteful back-recombination of electrons and holes, which is the principal enemy of artificial photochemical systems [9–10].

Two lessons from this biological blueprint are instructive for materials design. First, the photosensitization principle: Light harvesting and catalytic chemistry are performed by distinct, spatially separated components that are electronically coupled at their interface. The antenna extends the absorption, the reaction centre does the chemistry. Second, the spatial organisation principle: Efficient photochemistry requires the light-generated charge carriers to be separated at an interface and driven to specific catalytic sites before they recombine. Achieving these two principles in a synthetic, scalable, and inexpensive material is the central aspiration, if not the “holy grail” of artificial photosynthesis.

**Figure 2:**
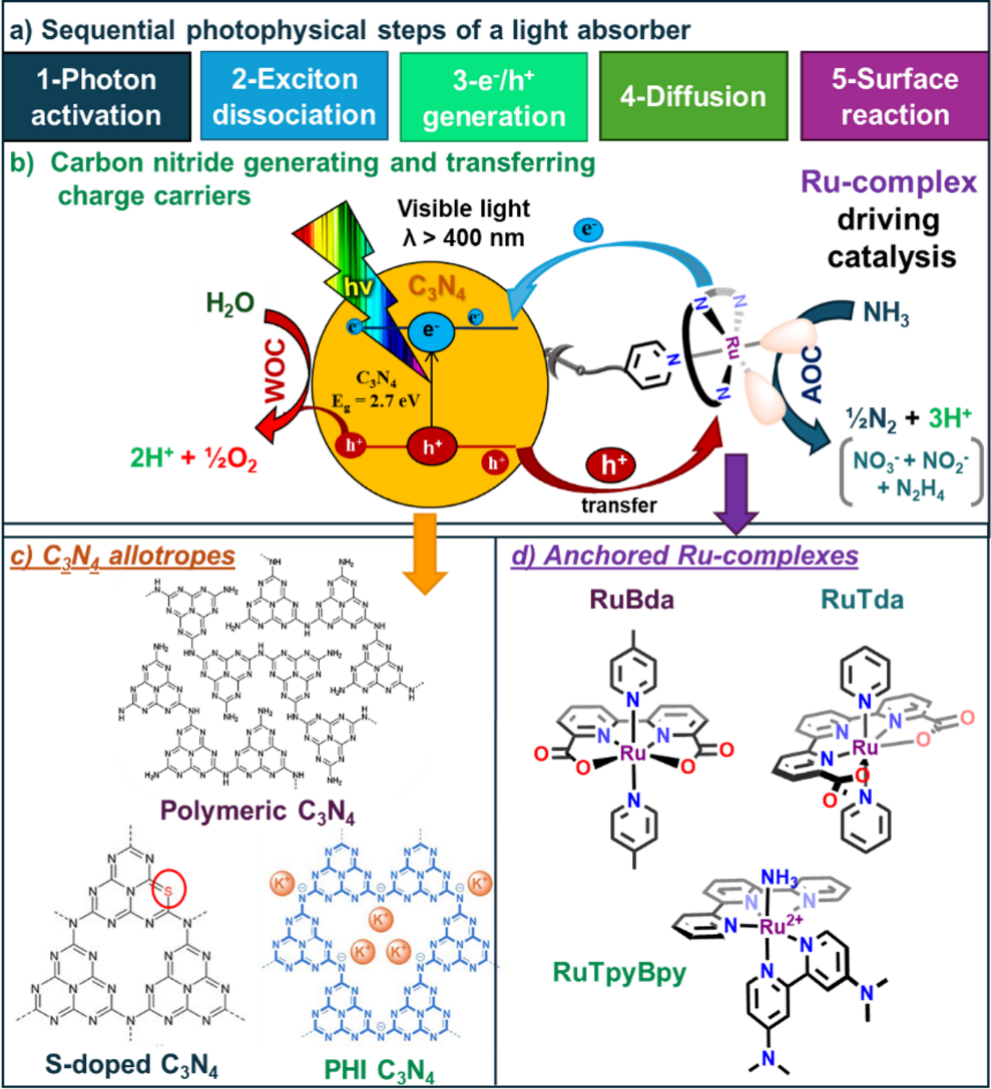
(a) Sequential photophysical and chemical steps of a catalytically active absorber, (b) illustrative scheme of the electron and hole transfer in the RuBda@C_3_N_4_ interface, (c) three C_3_N_4_ allotropes, and (d) three Ru-complexes.

In artificial photoelectrocatalytic systems, light-driven reactions follow five sequential steps (Figure 2a): (1) Photon absorption by the semiconductor generates a bound electron–hole pair, or exciton; (2) the exciton undergoes dissociation into spatially separated free charge carriers, a thermally and field-assisted step driven by the intrinsic electric field at the semiconductor–electrolyte junction; (3) the resulting free electrons populate the conduction band, while holes remain in the valence band; (4) carriers diffuse through the bulk toward the surface; and (5) the surface-bound molecular catalyst drives the target redox reaction. Here, either water oxidation or ammonia oxidation at the anchored Ru centre.

This distinction matters especially for C_3_N_4_. Unlike inorganic semiconductors with high dielectric constants, where exciton formation and dissociation are instantaneous, C_3_N_4_ has a higher exciton binding energy (0.1–0.4 eV [11]) that makes carrier separation a predominant rate-limiting step. The applied bias in a photoelectrochemical cell assists this dissociation by sweeping free carriers away from the recombination zone, an advantage that goes beyond simply reducing the thermodynamic requirement, and that is discussed further in section G.

### C) Carbon nitride as the semiconductor light absorber

C_3_N_4_ is a transformative organic semiconductor photocatalyst, which has, in fact, over the past decade, transformed into one of the benchmark photocatalysts [12–14]. It is synthesised by solid-state thermal polycondensation of cheap, abundant, nitrogen-rich organic precursors such as melamine, dicyandiamide, or urea at temperatures between 500 and 600 °C, a process requiring no solvents, no metal catalysts, and no complex equipment. The resulting material is a two-dimensional polymer composed of heptazine (tri-s-triazine) or s-triazine building block units linked by tertiary amines into extended graphitic sheets, with a bandgap of approximately 2.7 eV with optical absorption onset at 460 nm (Figure 2b), firmly within the visible range of the solar spectrum [12–14].

C_3_N_4_ is attractive not only for its multipurpose catalytic platform but also for its light absorption and the simultaneous combination of these properties. Its conduction band edge sits at approximately −1.1 V vs the normal hydrogen electrode (NHE), providing sufficient thermodynamic driving force for many reduction reactions. Its valence band edge at approximately +1.6 V vs NHE supports oxidation chemistry. It is chemically inert to most organic solvents and electrolytes, thermally stable in air up to around 600 °C, and biocompatible. Crucially for hybrid material design, the nitrogen-rich aromatic heptazines contain accessible functional groups, such as primary amines (–NH_2_) and secondary amines (=NH), thus providing handles for both covalent (e.g. –CO–NH– [15]) and non-covalent (e.g. π–π [16]) attachment of functional molecular guests/appendages [8,12,17].

The C_3_N_4_ family is not a single material but a rich structural landscape (Figure 2c). Polymeric C_3_N_4_ (Table 1), the most widely studied form, is semicrystalline, with moderate charge carrier mobility and a bandgap tunable between 2.6 and 2.8 eV, depending on synthesis conditions [12,18]. Poly(heptazine imide) (PHI, Table 1), synthesised in eutectic salt melts, is a highly crystalline ionic variant in which negatively charged nitrogen bridges compensate intercalated metal cations, yielding superior charge carrier mobility, reduced defect density, and extended exciton lifetimes that are of relevance to photoelectrocatalytic performance [19]. Amorphous sulfur-doped C_3_N_4_ (S-C_3_N_4_, Table 1), prepared from sulfur-containing precursors such as purpald, achieves a narrower bandgap of 1.8–2.0 eV and a higher surface area, accessing a larger fraction of the solar spectrum at the cost of somewhat reduced charge transport efficiency [13]. Each allotrope presents a distinct combination of optical, electronic, and surface properties, and their comparison in hybrid architectures is itself a source of mechanistic insight.

**Table 1:** Comparison of the key properties of C_3_N_4_-based materials.

Property	Polymeric C_3_N_4_	Crystalline PHI	Amorphous S-C_3_N_4_

structure	semicrystalline	highly ordered	disordered/distorted
bandgap [eV]	2.6–2.8	2.7	1.8–2.0
charge mobility	moderate	high	low
active centres	π–π, NH*_x_*	M^+^, N^-^, π–π, NH*_x_*	S atom, π–π, NH*_x_*
surface area	moderate: <50 m^2^·g^−1^	low: <30 m^2^·g^−1^	high: <100 m^2^·g^−1^
application focus	versatile, stable	high-performance	high surface reactivity

Beyond allotrope selection, post-synthetic strategies offer a complementary route to tuning the optical and surface properties of polymeric C_3_N_4_ without resynthesis. Thermal oxidation etching, acid treatment, and alkali-assisted exfoliation can shift the valence band edge, narrow the bandgap, and introduce surface defects that serve as additional anchoring sites or charge-trapping centres, further expanding the parameter space available for hybrid photoelectrode optimization [4–5].

From the perspective of the antenna analogy introduced in section B, C_3_N_4_ occupies the role of the photosensitizer in the hybrid system. It absorbs visible light, generates electron–hole pairs, and transports these charge carriers to the semiconductor surface where the molecular catalyst awaits [8]. Its medium bandgap means it can harvest blue and green photons that pass unused through many metal oxide photocatalysts. Its 2D layered structure, stabilised by π–π stacking interactions between heptazine sheets, provides a large, flat, aromatic surface ideally suited for the non-covalent adsorption of flat aromatic molecular catalysts through complementary π–π and C–H···π interactions [8,16].

### D) Ruthenium molecular catalysts: selectivity and multielectron chemistry

Ruthenium is suited for ammonia oxidation for three reasons. First, the six-electron, six-proton oxidation of NH_3_ to N_2_ requires a metal centre capable of cycling through multiple oxidation states sequentially, Ru(II) through Ru(V), within a narrow and accessible potential window (≈0.2–1.2 V vs NHE), accumulating the oxidizing equivalents needed for N–N bond formation without releasing oxidized intermediates such as hydrazine or hydroxylamine. Second, ammonia is a strong σ-donor that coordinates readily to Ru(II), positioning the substrate in the inner coordination sphere for successive proton-coupled electron-transfer steps. Third, the ligand environment around Ru can be rationally tuned to control the thermodynamics of each Ru(*n*)/Ru(*n* + 1) transition, the NH_3_/H_2_O binding selectivity, and the geometry of the high-valence Ru=NH intermediate that precedes N–N coupling. No first-row transition metal combines oxidation state accessibility, substrate affinity, and ligand tunability with the same reliability, making Ru-based molecular catalysts the benchmark in water oxidation catalysis (WOC) and ammonia oxidation catalysis (AOC).

If C_3_N_4_ is the antenna and the charge separator, the molecular Ru-complex is the reaction centre, the component that accepts holes from the semiconductor valence band and uses their oxidising power to drive the multielectron, multiproton transformation of the substrate. Ruthenium occupies a privileged position in oxidation catalysis because of its unique ability to access a wide range of stable oxidation states (Ru(II), Ru(III), Ru(IV), Ru(V), and even Ru(VI) under forcing conditions) within a relatively narrow potential window, and because the ligand field around the ruthenium centre can be finely tuned to control the thermodynamics and kinetics of each oxidation step [20–21].

Three benchmark Ru catalysts are particularly relevant to the proposed hybrid strategy (Figure 2d). RuBda (Ru coordinated by 2,2′-bipyridine-6,6′-dicarboxylic acid [22–23]) is the current state-of-the-art catalyst for WOC and AOC [24–25]. Its carboxylate arms provide both strong coordination to the metal and axial or equatorial ligands, which can be conveniently used for surface anchoring, and its low water oxidation onset potential of 1.1 V vs NHE is among the lowest reported for any molecular catalyst [22,24]. When non-covalently attached to carbon paper electrodes through C–H···π interactions, RuBda achieves a turnover number of 7500 and Faradaic efficiency of 100% for electrochemical ammonia oxidation in propylene carbonate, producing N_2_ as the sole nitrogen-containing product, a benchmark result that established the molecular framework for AOC [26]. RuTda (Ru coordinated by 2,2′:6′,2″-terpyridine-6,6″-dicarboxylic acid), with its extended terpyridine backbone, has been covalently anchored onto triazine-based covalent organic frameworks (COFs) by Llobet and co-workers, achieving photocatalytic water oxidation with a turnover number of 220 and demonstrating that molecular Ru catalysts retain their activity upon surface immobilisation in photochemical conditions [27]. RuTpyBpy (Ru coordinated by terpyridine and bipyridine), while exhibiting a higher overpotential for water oxidation, has been shown to produce nitrate (NO_3_^−^) selectively from ammonia oxidation in aqueous media when immobilised on cyclodextrin-functionalized ITO electrodes, illustrating that product selectivity in ammonia oxidation (N_2_ vs NO_3_^−^ vs N_2_H_4_) is tunable through catalyst structure and the reaction environment [28–29].

The mechanistic basis for this selectivity lies in two competing reaction pathways [21–22,30–31]. The unimolecular water/ammonia nucleophilic attack mechanism proceeds when a water or ammonia molecule attacks a high-valence Ru=O or Ru=NH intermediate, generating peroxo or hydrazido species that subsequently release O_2_ or N_2_ via a series of proton-coupled electron-transfer steps. The bimolecular intermolecular mechanism based on the interaction of two metal units (I2M) proceeds through the coupling of two high-valence Ru species to form a Ru–X–X–Ru (X = O, N) bridged intermediate, releasing O_2_ or N_2_ upon reductive elimination. Selectivity toward N_2_ over hydrazine is thermodynamic: Ammonia oxidation to N_2_ is more exergonic than hydrazine formation, which requires a very specific catalyst design to become competitive. Surface immobilisation further disfavours the bimolecular I2M pathway by geometrically constraining adjacent catalyst molecules, but this is a secondary kinetic reinforcement rather than the main selectivity determinant [26,32]. This mechanistic insight is relevant to the design of hybrid C_3_N_4_–Ru electrodes, where the surface density and spatial arrangement of the anchored catalyst molecules can be controlled through the choice of anchoring chemistry.

### E) Covalent and non-covalent anchoring: two routes to the heterointerface

The quality of the C_3_N_4_–Ru interface, the electronic coupling between the semiconductor and the molecular catalyst, the stability of the catalyst under operating conditions, and the catalyst’s accessibility to the substrate depend critically on how the Ru complex is grafted to the C_3_N_4_ surface. Two different strategies have been developed, each with distinct advantages and limitations [8,17].

Covalent anchoring exploits the amine and imine functional groups that decorate the surface of C_3_N_4_ because of incomplete polycondensation during synthesis [15]. These –NH_2_ groups react with activated carboxylic acid derivatives (acyl chlorides or active esters) to form stable amide bonds that link the carbon nitride surface covalently to a bifunctional linker molecule. The linker typically contains a terminal pyridine group at one end, an aromatic or aliphatic bridging moiety, and the reactive acyl group at the other end. Subsequently, a ruthenium precursor then coordinates to the pyridine nitrogen, completing the assembly. The resulting covalent connection is thermodynamically robust, surviving repeated electrochemical cycling and exposure to the reaction medium without significant catalyst leaching. A proof-of-concept, using preliminary unpublished data, to strengthen the credibility of this strategy, RuBda covalently grafted onto polymeric C_3_N_4_ nanoparticles, achieved a ruthenium surface density of 5.5 mol % Ru (5.5 × 10^−4^ mol per gram of C_3_N_4_), validated by ion-coupled plasma and a turnover frequency for ammonia oxidation of 5.6 s^−1^, surpassing by a few orders of magnitude the activity of the best non-covalently immobilized benchmark. Importantly, transferring the electrode to a fresh electrolyte and performing a rinse test confirmed that catalytic activity follows the electrode rather than dissolved species, confirming that the catalyst remained securely anchored without delamination.

Non-covalent anchoring takes an altogether different approach, exploiting the π-electron density of the aromatic heptazine surface rather than its reactive functional groups [16,32]. Ru complexes functionalized with planar aromatic pyrene groups can adsorb spontaneously onto the C_3_N_4_ surface through a combination of π–π stacking between the pyrene unit and the heptazine rings, and C–H···π interactions between the aliphatic protons of the complex periphery and the aromatic surface. This one-pot process, that is, dissolving the pyrene-functionalized Ru complex in a suitable solvent, adding the C_3_N_4_ support, allowing adsorption to reach equilibrium, and washing away excess complex, requires no reactive chemistry and no elevated temperatures, making it synthetically accessible even for complex, sensitive catalyst molecules. The non-covalent strategy has already produced a landmark result: A 15-mer RuTda oligomer non-covalently adsorbed onto C_3_N_4_ coated on FTO glass achieved a turnover number of 3000 and a turnover frequency of 0.4 s^−1^ for photoelectrocatalytic water oxidation, consuming only micrograms of ruthenium per gram of support, an atom efficiency figure that makes the use of a precious metal economically defensible [16]. Crystalline PHI is a promising support for non-covalent anchoring: Its highly ordered aromatic surface, free of the structural disorder that characterises amorphous C_3_N_4_, presents a geometrically regular array of π-stacking sites that can accommodate the flat pyrene anchor in a defined orientation, maximising electronic coupling between the catalyst and the semiconductor.

Covalent and non-covalent bonding are not competing alternatives but complementary tools. Covalent anchoring prioritises stability and provides a well-defined, short electron coupling pathway between C_3_N_4_ and the Ru centre. Non-covalent anchoring prioritises synthetic simplicity and atom efficiency and may be better suited to crystalline PHI, where surface reactivity toward nucleophilic chemistry is lower. Strategies comparison across the three C_3_N_4_ allotropes, polymeric, PHI, and S-doped amorphous, would reveal structure–activity relationships that cannot be obtained by studying each isolated allotrope.

A third interface strategy, yet to be explored in the C_3_N_4_–Ru context, is the direct coordination of isolated Ru atoms within the interlayer space of the C_3_N_4_ scaffold, that is, the single-atom catalyst (SAC) approach. Unlike covalent or non-covalent anchoring of a preformed complex, SACs position individual metal atoms at sub-nanometer proximity to the semiconductor surface, potentially maximizing electronic coupling between antenna and catalytic centre while eliminating the need for a discrete linker or pyrene-functionalized ligand. Open challenges include metal atom migration and agglomeration under prolonged irradiation, and the difficulty of distinguishing genuine single-atom activity from sub-nanometer cluster contributions under operando conditions [33]. A full mechanistic discussion lies beyond the scope of this perspective; SAC nonetheless represents a parallel, and potentially more direct, realization of the antenna–reaction-centre coupling principle that motivates the hybrid strategy described here.

A practical consideration that applies to all anchoring strategies is the photostability of the Ru complex under operating conditions. Ru polypyridyl complexes are among the most photostable molecular catalysts in the WOC and AOC literature [34], and ligand frameworks such as bda and tda are not expected to degrade under visible-light irradiation [15–16,33] The more likely degradation pathways are oxidative cleavage of the amide linker in covalently anchored systems, or displacement of the pyrene anchor by photogenerated reactive oxygen species in non-covalently adsorbed ones, both of which manifest as Ru leaching into solution. The rinse test reported for the RuBda–C_3_N_4_ covalent system (section E) confirms anchoring integrity under electrochemical conditions, but equivalent stability tests under sustained bandgap illumination remain to be reported and should be considered an essential benchmark, alongside ICP quantification of Ru leaching and post-catalysis XPS, as demonstrated for analogous Ru(bda)-based electrodes by X-ray absorption spectroscopy after extended bulk electrolysis [26].

### F) Why ammonia oxidation?

Ammonia offers compelling thermodynamic advantages as a hydrogen carrier. Its oxidation requires significantly less energy than water splitting, only 33 MJ per kg H_2_ at −0.092 V vs NHE, compared to 180 MJ per kg H_2_ at 1.23 V vs NHE for the oxygen evolution reaction, representing a substantially lower thermodynamic barrier. Combined with its high volumetric energy density, liquid ammonia stores 1.9 times more energy per liter than liquid hydrogen, making it an attractive medium for carbon-free energy storage and transport [35].

### G) The case for photoelectrocatalysis: combining light and bias

Photocatalysis and electrocatalysis each address part of the energy challenge of driving thermodynamically uphill reactions [29,36]. Pure photocatalysis relies entirely on photon energy to supply the thermodynamic driving force, imposing strict requirements on the semiconductor band positions relative to the reaction redox potentials, and offering limited kinetic control. Pure electrocatalysis applies an external potential to drive the reaction, offering precise control of potential but consuming electrical energy that must itself be generated cleanly. Photoelectrocatalysis [19,37], the simultaneous application of light illumination and an electrochemical bias to a semiconductor photoelectrode, combines the advantages of both: Photon energy reduces the required applied potential (the photovoltage generated by the semiconductor partially compensates the thermodynamic requirement of the reaction), while the applied bias provides independent kinetic control over charge carrier dynamics, suppresses recombination by sweeping carriers away from the semiconductor bulk, and enables product selectivity to be tuned by adjusting the electrode potential.

Photoelectrocatalytic ammonia oxidation offers an unexplored and underappreciated advantage. Ammonia oxidation requires the removal of six electrons and six protons (for the N_2_ pathway) or eight electrons and nine protons (for the NO_3_^−^ pathway), a multistep process in which each intermediate must be stabilised long enough to undergo the next oxidation event [25,29]. The applied bias in a photoelectrochemical cell keeps the electrode potential within the window required for sequential Ru oxidation (Ru(II)→Ru(III)→Ru(IV)→Ru(V)), while the photogenerated holes from C_3_N_4_ provide additional oxidising equivalents without requiring the full thermodynamic cost to be met by the external power supply. In this direction, the C_3_N_4_–Ru hybrid photoelectrode is the synthetic realisation of the Photosystem II concept: Light provides the energy, the semiconductor separates the charges, and the molecular catalyst, precisely positioned at the semiconductor surface through covalent or non-covalent anchoring, does the multielectron bond-breaking chemistry [9].

Despite this compelling conceptual foundation, and despite the availability of all the necessary components, including (a) photoactive C_3_N_4_ semiconductors in multiple allotropic forms, (b) well-characterised molecular Ru catalysts with demonstrated AOC activity, and (c) established anchoring strategies that preserve catalyst activity upon immobilisation, no study has yet reported the photoelectrocatalytic oxidation of ammonia using a Ru-functionalized C_3_N_4_ photoelectrode. The closest precedents are the photoelectrocatalytic water oxidation activity of RuTda oligomers on C_3_N_4_/FTO [16], which confirms the compatibility of the hybrid architecture with the PEC configuration, and the electrocatalytic ammonia oxidation activity of RuBda on carbon paper [26], which confirms that molecular Ru catalysts retain their AOC function upon surface anchoring. The intersection of these two lines of evidence, photoelectrocatalytic AOC at a C_3_N_4_–Ru hybrid electrode, has not been crossed.

## Outlook

The combination of C_3_N_4_ semiconductors with molecular Ru catalysts through covalent and non-covalent anchoring strategies offers a chemically rational, modern, accessible, and mechanistically transparent route to artificial photosynthetic systems for solar-driven ammonia oxidation, though known and implemented in WOC. Inspired by the design principles of natural photosynthesis, photosensitization, spatial charge separation, and the coupling of a light-harvesting antenna to a multielectron catalytic centre, this hybrid approach addresses the fundamental gap between homogeneous and heterogeneous catalysis by using molecular selectivity and semiconductor light harvesting in the same interfacial architecture. The allotropic diversity of C_3_N_4_, from semi-crystalline polymeric forms over highly ordered PHI to high-surface-area amorphous S-doped variants, provides a tunable platform for exploring how semiconductor structure controls charge carrier dynamics and thereby catalytic performance.

The step forward is clear in its direction if not yet in its details: It includes fabrication and characterisation of C_3_N_4_–Ru hybrid photoelectrodes across varying allotropes, Ru catalyst structures, and anchoring chemistries, tested under simultaneous illumination and electrochemical bias in the photoelectrocatalytic ammonia oxidation. That no such study has yet been published is a gap in the literature. The materials components are available, the conceptual framework is established, and the precedent reactions (WOC and electrochemical AOC) have validated the system’s key feasibility. Testing them together in photoelectrocatalysis is the experiment that this perspective envisages building toward.

## Data Availability

Data sharing is not applicable to this article as no new experimental datasets were generated. The discussion draws primarily from previously published studies, which are cited accordingly. Two sets of preliminary unpublished results are included to support the proposed strategy; the underlying data are available from the corresponding authors upon reasonable request and will be reported in full in a forthcoming publication.
